# *Convolvulus althaeoides* as a Potential Natural Source in Cancer Prophylaxis and Treatment: LC‐ESI‐MS/MS, GC‐MS, In Vitro, and In Silico Investigations

**DOI:** 10.1002/cbdv.71792

**Published:** 2026-09-30

**Authors:** Ahmed R. Hassan, Shaimaa Negm El‐Dein, Amany M. Korkor, Nourelhoda F. Hassan, Ahmed A. M. A. Selim

**Affiliations:** ^1^ Medicinal and Aromatic Plants Department Desert Research Center Cairo Egypt; ^2^ Department of Pharmacognosy Faculty of Pharmacy (Girls) Al‐Azhar University Cairo Egypt

**Keywords:** anticancer activity, *Convolvulus althaeoides*, GC‐MS, in silico study, LC‐ESI‐MS/MS

## Abstract

The current study aims to assess the potential relevance of the successive fractions, *n*‐hexane and ethyl acetate (EtOAc) of *Convolvulus althaeoides* in cancer management. Our study demonstrated significant in vitro cytotoxic activity with IC_50_ values of 5.8, 2.5, and 2.8 for EtOAc and 7.1, 5.3, and 5.3 µg/mL for *n*‐hexane against HepG‐2, MCF‐7, and HCT‐116, respectively, comparable to doxorubicin (4.8, 5.6, and 6.5 µg/mL), with a higher IC_50_ against normal human cell line. EtOAc and *n*‐hexane fractions exhibited substantial antioxidant using DPPH (97.22% and 90.12%, respectively) and moderate anti‐inflammatory properties by HRBC method (34.95% and 41.9%, respectively). LC‐ESI‐MS/MS analysis of EtOAc fraction revealed 41 peaks with varied chemical structures, indicating its richness of phenolic compounds. GC‐MS of *n*‐hexane fraction indicated an abundance of noteworthy fatty acids, phytol, and phytosterols. The in silico results showed good pharmacophore features and docking scores for some of the identified compounds on ERR‐3 (quercetin‐3‐*O*‐pentoside −11.497), microtubule (rutin −10.801), and tubulin (quercetin‐3‐*O*‐glucoside −7.113).

## Graphical abstract

*n*‐hexane and EtOAc fractions of *Convolvulus althaeoides* aerial parts have potent cytotoxicity, significant antioxidant, and moderate anti‐inflammatory effects. These activities emphasize the potential role of this plant in cancer prophylaxis and treatment. LC‐ESI‐MS/MS and GC‐MS analyses show the chemical profile advantages responsible for these activities, supported by in silico studies.

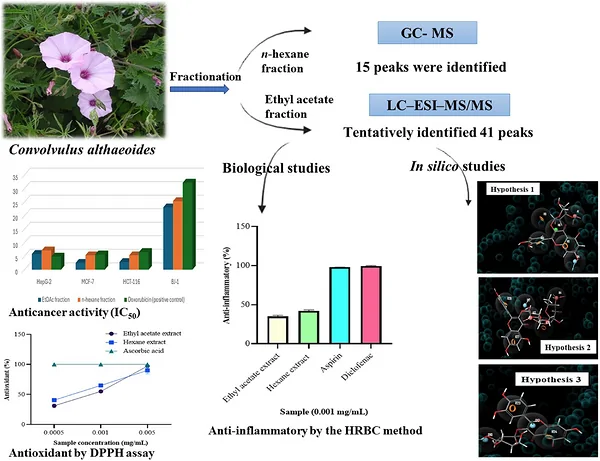

## 1 Introduction

Cancer, characterized by uncontrolled cell proliferation that results in tissue damage and may ultimately lead to death, remains one of the most significant health challenges worldwide. The development of cancer involves multiple molecular pathways and various triggering factors that transform normal cells into malignant ones. These factors include DNA damage induced by oxidative stress and chronic inflammation, both of which promote the release of inflammatory mediators that facilitate tumor growth and progression [1, 2]. The complexity of these mechanisms has made it difficult for a single therapeutic approach to effectively prevent or cure the disease. Despite the availability of numerous anticancer agents that have improved patient management, many types of cancer continue to exhibit limited or no response to current treatment modalities. Cancer prevention strategies primarily focus on minimizing exposure to carcinogenic factors, including oxidative stress and chronic inflammation. In contrast, therapeutic approaches rely on a combination of surgery, radiotherapy, chemotherapy, and more advanced modalities such as hormone therapy and antibody‐based treatments. Chemotherapy commonly involves the use of multiple agents that target different molecular pathways implicated in carcinogenesis. Some chemotherapeutic drugs exert their effects by directly damaging DNA, whereas others interfere with DNA or RNA synthesis or inhibit essential enzymes required for cell division [3, 4, 5].

Plant‐derived compounds play a significant role in both the prevention and treatment of cancer. For example, polyphenolic compounds exhibit potent antioxidant and anti‐inflammatory properties, fatty acids can induce apoptosis, and *Vinca* alkaloids and taxanes inhibit cancer cell proliferation by stabilizing microtubules and causing cell cycle arrest. Moreover, plant extracts offer the advantage of containing diverse bioactive constituents that can simultaneously target multiple molecular pathways and carcinogenic triggers involved in cancer development [6, 7, 8, 9].

*Convolvulus althaeoides* (mallow bindweed) is a perennial flowering species belonging to the Convolvulaceae family and is widely distributed throughout the Mediterranean region [10]. Although the *Convolvulus* genus has been extensively recognized for its broad spectrum of biological activities, including cytotoxic effects [11, 12, 13], previous investigations on *C. althaeoides* have mainly focused on its volatile constituents and essential oil composition [14, 15, 16]. Notably, one study demonstrated that the essential oil of *C. althaeoides* exhibited strong cytotoxic activity against the human breast cancer cell line (MCF‐7), with an IC_50_ = 8.16 µg/mL [14].

Based on the aforementioned findings, the present study aimed to investigate the potential of two fractions obtained from the 70% ethanolic extract of the aerial parts of *Convolvulus althaeoides*: the *n*‐hexane fraction, which is enriched in volatile constituents, and the ethyl acetate (EtOAc) fraction, which contains the majority of the plant's phenolic compounds. The study evaluated their potential roles in cancer prevention through antioxidant and anti‐inflammatory activities, as well as their therapeutic potential through cytotoxic activity against cancer cells. The phytochemical profiles of the two fractions were characterized using GC–MS analysis for the *n*‐hexane fraction and LC–ESI–MS/MS analysis for the EtOAc fraction. In addition, the possible molecular mechanisms underlying the cytotoxic effects of the identified constituents were explored using in silico approaches, including pharmacophore modeling and molecular docking studies.

## 2 Results and Discussion

### 2.1 LC‐ESI‐MS/MS Profile of EtOAc Fraction From *C. althaeoides* Aerial Parts

Comprehensive phytochemical characterization of the ethyl acetate (EtOAc) fraction obtained from the aerial parts of *Convolvulus althaeoides* was performed using LC–ESI–MS/MS analysis in the negative ionization mode. A total of 41 chromatographic peaks, representing structurally diverse metabolites, were tentatively identified (Table 1 and Figure 1). Compound annotation was based on retention times (*t*_R_), observed molecular ion peaks, and characteristic fragmentation patterns (Figures S1–S41), with further confirmation through comparison with published literature and mass spectral databases. The identified metabolites predominantly comprised twelve flavonoid‐related compounds, including two flavonols, nine flavonol *O*‐glycosides, and one coumarin derivative. Additionally, twenty‐four phenolic acids and their derivatives were detected, together with two sugar alcohols, two fatty acids, and one α‐hydroxy carboxylic acid, quinic acid, which is commonly distributed in plant species. The fundamental chemical structures of selected phytochemicals identified in the EtOAc fraction of *C. althaeoides* are presented in Figure 2.

**TABLE 1:** Chemical profile (LC‐ESI‐MS/MS) of the active constituents in ethyl acetate (EtOAc) fraction of *C. althaeoides* aerial parts.

Peak No.	*t*_R_ (min)	Peak area (%)	(M−H)^−^ *m/z*	Fragments (MS/MS)	Tentative identification	Reference
1	2.22	3.45	191	173, 171, 127, 109	Quinic acid	[10, 12, 17, 18]
2	2.28	0.70	135	135	2‐methyl erythritol	[19]
3	2.35	0.26	151	107, 100	Xylitol	[19]
4	2.86	0.94	353	191, 173, 135	Caffeoylquinic acid (Chlorogenic acid)	[10, 12, 19, 21]
5	3.33	0.17	353	191, 173, 135	Caffeoylquinic acid isomer	[10, 12, 19, 21]
6	3.78	0.27	487	353, 191, 161	Caffeoylquinic acid‐*O*‐xylitol	[10, 12, 19]
7	4.03	1.21	487	353, 191, 161	Caffeoylquinic acid‐*O*‐xylitol isomer	[10, 12, 19]
8	4.26	0.22	341	179, 161, 135	Caffeic acid‐*O*‐hexoside	[10, 20]
9	4.47	0.33	341	179, 161, 135	Caffeic acid‐*O*‐hexoside isomer	[10, 20]
10	4.69	0.47	313	179, 161, 135	Caffeic acid‐*O*‐xylitol	[19]
11	4.91	0.59	313	179, 161, 135	Caffeic acid‐*O*‐xylitol isomer	[19]
12	5.98	2.21	471	353, 191, 179, 161, 135	Caffeoylquinic acid‐*O*‐erythritol‐2‐methyl	[10, 12, 19]
13	6.22	2.11	471	353, 191, 179, 161, 135	Caffeoylquinic acid‐*O*‐erythritol‐2‐methyl isomer	[10, 12, 19]
14	6.44	1.92	471	353, 191, 179, 161, 135	Caffeoylquinic acid‐*O*‐2‐methyl erythritol isomer	[10, 12, 19]
15	7.55	1.72	515	353, 191, 179, 173, 135	Dicaffeoylquinic acid (3,5‐di‐*O*‐caffeoylquinic acid)	[10]
16	7.76	3.05	515	353, 191, 179, 173, 135	3,5‐di‐*O*‐caffeoylquinic acid	[10]
17	8.19	4.12	515	353, 203, 191, 179, 173, 135	4,5‐di‐*O*‐caffeoylquinic acid	[10]
18	8.38	3.42	515	353, 335, 203, 191, 179, 173, 135	3,4‐di‐*O*‐caffeoylquinic acid	[12, 19, 23]
19	8.52	4.34	609	301	Quercetin‐*O*‐rhamnosyl‐hexoside (Rutin)	[10, 12, 19, 30]
20	8.67	6.34	463	301	Quercetin‐*O*‐hexoside (Quercetin‐3‐*O*‐galactoside)	[12, 20, 23]
21	8.94	7.20	463	301	Quercetin‐*O*‐hexoside isomer	[12, 20, 23]
22	9.15	3.57	463	301	Quercetin‐*O*‐hexoside isomer	[12, 20, 23]
23	9.36	3.49	463	301	Quercetin‐*O*‐hexoside isomer	[12, 20, 23]
24	9.42	1.16	161	133, 117, 105	Umbelliferone	[31]
25	9.54	1.50	433	301	Quercetin‐*O*‐pentoside (Quercetin‐3‐*O*‐arabinoside)	[12, 21]
26	9.73	0.65	433	301	Quercetin‐*O*‐pentoside isomer	[12, 21]
27	9.95	0.83	417	285, 255, 227	Kaempferol‐*O*‐pentoside	[12, 19]
28	10.12	4.23	649	515, 353, 191, 173, 135	Dicaffeoylquinic acid‐*O*‐xylitol	[10, 19]
29	10.28	5.02	327	291, 229, 211, 183, 171	Trihydroxy‐10,15‐octadecadienoic acid	[12, 19, 20, 23]
30	10.50	4.38	771	609, 301	Quercetin‐*O*‐rhamnosyl hexosyl‐hexoside (Rutin*‐O*‐hexoside)	[10, 12, 19, 30]
31	10.63	2.75	633	515, 353, 191, 179, 173, 135	Dicaffeoylquinic acid‐*O*‐erythritol‐2‐methyl	[10, 19]
32	10.97	2.35	329	229, 211, 183, 171, 139, 127	Trihydroxy‐10‐octadecenoic acid	[12, 19, 20, 23]
33	11.92	1.39	431	179, 161, 133	Caffeic acid derivative	
34	11.98	0.69	193	161, 133	Ferulic acid	
35	12.14	1.63	431	179, 161, 133	Caffeic acid derivative	
36	12.41	0.83	301	229, 179, 151, 121, 107	Quercetin	[10, 24, 30]
37	12.54	3.46	445	179, 161, 133	Caffeic acid derivative	
38	12.77	0.77	445	327, 179, 161, 133	Caffeic acid derivative	
39	12.83	0.87	423	179, 161, 133	Caffeic acid derivative	
40	13.10	2.39	329	314, 299, 271	Dimethyl‐*O*‐ellagic acid (3,3'‐di‐*O*‐methyl ellagic acid)	[25]
41	13.29	1.95	285	229, 185	Kaempferol	[10, 24, 30]

**FIGURE 1:**
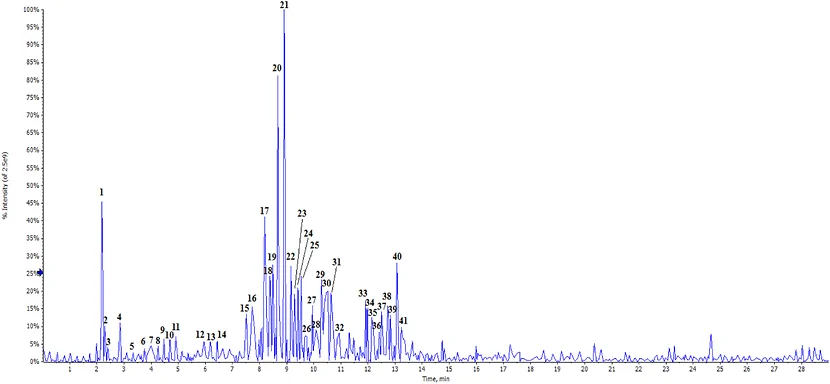
LC‐ESI‐MS‐MS base peak intensity (BPI) chromatogram in the negative mode of the EtOAc fraction from *C. althaeoides* aerial parts.

**FIGURE 2:**
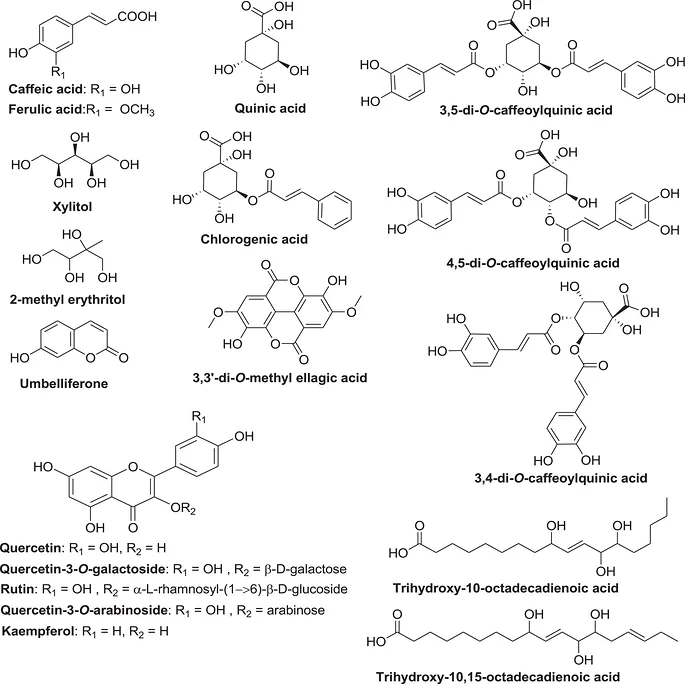
Chemical structures of some assigned phytochemicals in *C. althaeoides* EtOAc fraction.

The first peak (peak **1**; *t*_R_ = 2.22 min) displayed a molecular ion [M−H]^−^ at *m*/*z* 191 together with a characteristic fragment ion at *m/z* 173 [M−H−H_2_O]^−^, leading to its tentative identification as quinic acid [10, 12, 17, 18]. Quinic acid is a well‐established biosynthetic precursor of numerous phenolic metabolites, particularly hydroxycinnamic acids such as caffeic acid. In contrast, peaks **2** and **3** (*t*_R_ = 2.28 and 2.35 min, respectively) were tentatively assigned as the sugar alcohols 2‐methyl‐1,2,3,4‐butanetetrol (2‐methylerythritol) and xylitol, respectively. These assignments were based on their deprotonated molecular ions [M−H]^−^ observed at *m/z* 135 and 151, in agreement with previously reported data [19].

Phenolic acids and their derivatives constituted the largest group of metabolites identified in the EtOAc fraction, accounting for twenty‐four tentatively annotated peaks. Among these, peak **34** (*t*_R_ = 11.98 min) displayed a pseudo‐molecular ion [M−H]^−^ at *m/z* 193, which is characteristic of ferulic acid [10, 12, 20]. Peaks **4** and **5** (*t*_R_ = 2.86 and 3.33 min, respectively) were tentatively assigned as caffeoylquinic acid (chlorogenic acid) and one of its positional isomers. Both compounds displayed the same precursor ion at *m/z* 353 [M−H]^−^ and generated identical fragment ions at *m/z* 191, 179, 173, 135, although the relative abundances of these fragment ions differed, enabling their distinction [10, 12, 19, 21]. Likewise, peaks **15**, **16**, **17**, and **18** (*t*_R_ = 7.55, 7.76, 8.19, and 8.38 min, respectively) were pinpointed as di‐*O*‐caffeoylquinic acid isomers based on their common deprotonated molecular ion [M−H]^−^ at *m/z* 515 [10]. The discrimination of these isomeric compounds relied primarily on their MS/MS fragmentation patterns. Specifically, a predominant fragment ion at *m/z* 191 was indicative of 5‐caffeoyl derivatives, whereas a base peak at *m/z* 179 suggested the presence of 3‐caffeoyl derivatives [22]. In contrast, a dominant fragment ion at *m/z* 173 was considered diagnostic for 4‐caffeoyl derivatives. Peaks **15** and **16** exhibited identical MS/MS fragmentation patterns, producing product ions at *m/z* 353, 191, 179, 173, and 135. Both spectra were dominated by a base peak at *m/z* 191, followed by a second high‐intensity fragment at *m/z* 179. Based on these characteristic fragmentation features, both peaks were tentatively assigned as 3,5‐di‐*O*‐caffeoylquinic acid isomers. In contrast, peak **17** displayed a dominant fragment ion at *m/z* 173, suggesting its tentative identification as 4,5‐di‐*O*‐caffeoylquinic acid. Peak **18** was putatively characterized as 3,4‐di‐*O*‐caffeoylquinic acid, as evidenced by its fragment ions at *m/z* 353, 335, 203, 191, 179, 173, and 135. The predominance of the fragment ion at *m/z* 173, together with the second most intense ion at *m/z* 179, further supported this assignment [12, 19, 23]. Peaks **8** and **9** were recognized as caffeic acid‐*O*‐hexoside [10, 20]. Both compounds exhibited a deprotonated molecular ion [M−H]^−^ at *m/z* 341 and generated characteristic fragment ions at *m/z* 179 [M‐H‐162]¯, 161 [M−H−180]^−^, and 135 [M−H−180−CO_2_]¯. These fragmentation patterns are consistent with the neutral loss of a hexose moiety (162 amu), followed by the elimination of the caffeic acid moiety (180 amu), supporting the proposed structural assignments [24]. For peaks **6**, **7**, **10**, **11**, and **28** (*t*_R_ = 3.78, 4.03, 4.69, 4.91, and 10.12 min, respectively), a characteristic neutral loss of 134 amu was observed, indicating the presence of a xylitol moiety as a structural subunit of the corresponding metabolites [19]. Accordingly, peaks **6** and **7** were tentatively identified as caffeoylquinic acid‐*O*‐xylitol [10, 12, 19]. These compounds exhibited deprotonated molecular ions [M−H]^−^at *m/z* 487 and produced characteristic fragment ions at *m/z* 353 [M−H−134]^−^, 191 [M−H−134−162]^−^, and 161 [M−H−134−192]^−^. In contrast, peaks **10** and **11** revealed a molecular ion [M−H]^−^ at *m/z* 313, which fragmented to yield ions at *m/z* 179 [M−H−134]^−^, 161 [M−H−134−H_2_O]^−^, and 135 [M−H−134−CO_2_]^−^. These fragmentation characteristics supported their tentative identification as caffeic acid‐*O*‐xylitol derivatives [19]. Peak **28** was putatively identified as dicaffeoylquinic acid‐*O*‐xylitol [10, 19], based on its [M−H]^−^ at *m/z* 649 and the diagnostic product ions observed at *m/z* 515 [M−H−134]^−^, 353 [M−H−134−162]^−^, 191 [M−H−134−162−162]^−^, 173 [M−H−134−162−162−H_2_O]^−^, and 135. Conversely, peaks **12**, **13**, **14**, and **31** (*t*_R_ = 3.78, 4.03, 4.69, 4.91, and 10.12 min, respectively) were characterized by a neutral loss of 118 amu, which is indicative of the presence of a 2‐methylerythritol moiety as a structural component of the proposed metabolites [19]. This diagnostic fragmentation pattern supported the tentative assignment of these compounds as 2‐methylerythritol‐containing phenolic derivatives. Consequently, peaks **12**, **13**, and **14** were assigned as isomeric forms of caffeoylquinic acid‐*O*‐(2‐methylerythritol) or caffeoylquinic acid‐*O*‐erythritol‐2‐methyl [10, 12, 19]. These metabolites yielded pseudo‐molecular ions [M−H]^−^ at *m/z* 471, and generated characteristic fragment pattern at *m/z* 353 [M−H−118]^−^, together with a predominant base peak at *m/z* 191 [M−H−118−162]^−^. Additional diagnostic fragment ions were detected at *m/z* 179 [M−H−118−174]^−^, 161 [M−H−118−192]^−^, and 135 [M−H−118−174−CO_2_]^−^ supporting their proposed structural assignments. Similarly, peak **31** displayed its [M−H]^−^ at *m/z* 633 combined with characteristic fragments at *m/z* 515 [M−H−118]^−^, 353 [M−H−118−162]^−^, 191 [M−H−118−162−162]^−^, 179 [M−H−118−162−174]^−^, 173 [M−H−118−162−162−H_2_O]^−^, and 135 [M−H−118−162−174−CO_2_]^−^. These fragmentation features were consistent with the tentative identification of this compound as dicaffeoylquinic acid‐*O*‐erythritol‐2‐methyl [10, 19]. In addition, peaks **33**, **35**, **37**, **38**, and **39** (*t*_R_ = 11.92, 12.14, 12.54, 12.77, and 12.83 min, respectively) appeared to be derivatives of caffeic acid. Their proposed identities were supported by deprotonated molecular ions [M−H]^−^ at *m/z* 431, 431, 445, 445, and 423, respectively, as well as the presence of the characteristic product ions at *m/z* 179, 161, and 133. Finally, peak **40** (*t*_R_ = 13.10 min) afforded [M−H]^−^ at *m/z* 329 and fragments at *m/z* 314, 298, 271, 277, which is characteristic of dimethyl‐*O*‐ellagic acid (3,3'‐di‐*O*‐methyl ellagic acid) [25].

Overall, caffeoylquinic acids and their derivatives represented the predominant class of phenolic acids detected in the EtOAc fraction of *C. althaeoides*. These compounds are widely distributed among numerous plant species, including several members of the *Convolvulus* species [10, 12, 19]. Moreover, these compounds exhibit a broad spectrum of biological activities, including antioxidant, anticancer, anti‐inflammatory, antibacterial, antiviral, antidiabetic, and neuroprotective effects [26, 27, 28, 29].

Twelve peaks were tentatively assigned to flavonoid constituents. Among them, flavonol glycosides represented the predominant subclass detected in the EtOAc fraction of *C. althaeoides*, mainly comprising quercetin and kaempferol derivatives, together with their corresponding aglycones. The flavonol aglycones were detected as peaks **36** (*t*_R_ = 12.41 min) and **41** (*t*_R_ = 13.29 min), which exhibited deprotonated molecular ions [M−H]^−^ at *m/z* 301 and 285, respectively, resulting in the typical fragmentation pattern of quercetin and kaempferol, respectively [10, 24, 30]. The majority of the remaining flavonoid peaks were tentatively identified as quercetin glycosides. Specifically, peaks **20**, **21**, **22**, and **23** (*t*_R_ = 8.67, 8.94, 9.15, and 9.36 min, respectively) displayed identical deprotonated molecular ions [M−H]^−^ at *m/z* 463 and yielded the characteristic quercetin aglycone fragment at *m/z* 301 [M−H−162 (hexose unit)]^−^ following the neutral loss of a hexose residue (162 amu). Accordingly, these compounds were assigned as isomeric forms of quercetin‐*O*‐hexoside [12, 20, 23]. Based on previously reported data, one of these isomers was proposed to be quercetin‐3‐*O*‐galactoside (hyperoside) [10]. Likewise, peaks **25** (*t*_R_ = 28.30 min) and **26** (*t*_R_ = 28.30 min) were recognized as quercetin‐*O*‐pentoside isomers [12, 21], evidenced by exhibiting its [M−H]^−^ ion at *m/z* 433 and produced the diagnostic fragment ion at *m/z* 301 [M−H−132 (pentose unit)]^−^. Comparison with previous reports further suggested that one of these peaks may correspond to quercetin‐3‐*O*‐arabinoside [10]. Furthermore, mass spectrum for peaks **19** (*t*_R_ = 8.52 min) and **30** (*t*_R_ = 10.50 min) showed molecular ion signals [M−H]^−^ at *m/z* 609 and 771, respectively, together with the characteristic quercetin aglycone fragment at 301. In addition, peak **30** generated a fragment ion at *m/z* 609 [M−H−162 (hexose unit)]^−^. Based on these fragmentation characteristics, peaks **19** and **30** were tentatively assigned as quercetin‐*O*‐rhamnosyl‐hexoside (rutin) and quercetin‐*O*‐rhamnosyl hexosyl‐hexoside (rutin*‐O*‐hexoside), respectively [10, 12, 19, 30]. Peak **27** (*t*_R_ = 9.95 min) displayed a deprotonated molecular ion at *m/z* 417 [M−H]^−^, and yielded a predominant fragment ion at at *m/z* 285 [M−H−132]^−^ due to loss of pentoside moiety. Based on this characteristic fragmentation pattern, the compound was tentatively identified as kaempferol‐*O*‐pentoside [12, 19]. Finally, peak **24** (*t*_R_ = 9.42 min) exhibited mass fragment patterns of *m/z* 161 [M−H]^−^, 133 [M−H−CO]^−^, 117 [M−H−CO_2_]^−^, and 105, indicating a possible attribution to umbelliferone (7‐hydroxycoumarin) [31], which has previously been detected in *C. althaeoids* [10].

Overall, the obtained results indicate that quercetin derivatives constitute the predominant flavonoid subclass in *C. althaeoides*. Among them, quercetin‐*O*‐hexoside, hyperoside, and rutin were identified as the major flavonoid constituents of the EtOAc fraction, as summarized in Table 1 and Figure 1. are well recognized for their broad spectrum of pharmacological activities, e.g., antioxidant, antitumor, anti‐inflammatory, antibacterial, antiallergic, hypolipidaemic, antispasmodic, antiviral, antiulcerogenic, cardioprotective, neuroprotective, and antihypertensive [12, 32, 33, 34, 35, 36].

Additional metabolites were also tentatively identified in the EtOAc fraction of *C. althaeoides*. Specifically, peaks **29** (*t*_R_ = 10.28 min) and **32** (*t*_R_ = 10.97 min) gave [M−H]^−^ at *m/z* 327 and 329, respectively and produced major fragments at *m/z* 291, 229, 211, 183, 171 which lead to the tentative characterization of these two fatty acids as trihydroxy‐10,15‐octadecadienoic acid and trihydroxy‐10‐octadecenoic acid, respectively which relative to glycolipids [12, 19, 20, 23].

To our knowledge, comparison of the present findings with previously published reports on *C. althaeoides* [10], revealed that the compounds corresponding to peaks **2**, **3**, **6**‐**7**, **10**‐**11**, **12**‐**14**, **27**, **28**, **29**, **30**, **31**, **32**, and **40** haven't been previously reported in extracts of this species. Nevertheless, the metabolites represented by peaks **2**, **3**, **27**, **29**, and **32** have previously been identified in another member of the genus, *C. arvensis* [12, 19].

### 2.2 GC‐MS Analysis of *C. althaeoides n*‐Hexane Fraction

GC–MS analysis of the *n*‐hexane fraction of *C. althaeoides* enabled the identification of fifteen volatile constituents, corresponding to fifteen chromatographic peaks (Table 2; Figure 3). Among the identified metabolites, nine were classified as fatty acids and were detected in the saponifiable fraction. These included two unsaturated fatty acids, oleic, and linolenic acids, together with seven saturated fatty acids, namely palmitic, stearic, behenic, erucic, arachidic, tricosanoic, and heneicosanoic acids. Linolenic acid (39.30%) and palmitic acid (38.64%) were the predominant fatty acids, followed by stearic acid (9.23%), oleic acid (5.56%), and behenic acid (3.77%).

**TABLE 2:** Chemical profiling of *n*‐hexane fraction from *C. althaeoides* by GC‐MS.

Peak no.	RT (min)	Name of the compound	Molecular formula	MW *m/z*	Peak area (%)
Saponifiable fraction (Fatty acids)					
1	26.257	Palmitic acid	C_16_H_32_O_2_	200	38.64
2	31.663	Stearic acid	C_18_H_36_O_2_	256	9.23
3	32.497	Oleic acid	C_18_H_34_O_2_	356	5.56
4	36.327	Linolenic acid	C_18_H_30_O_2_	268	39.3
5	36.756	Arachidic acid	C_20_H_40_O_2_	270	1.36
6	39.185	Heneicosanoic acid	C_21_H_42_O_2_	326	0.22
7	41.526	Behenic acid	C_22_H_44_O_2_	340	3.77
8	42.111	Erucic acid	C_22_H_42_O_2_	338	1.66
9	43.812	Tricosanoic acid	C_23_H_46_O_2_	354	0.25
Unsaponifiable fraction (Silylated derivatives)					
10	14.222	1‐Hexadecanol, TMS derivative	C_19_H_42_OSi	314	0.31
11	16.369	Phytol, TMS derivative	C_23_H_48_OSi	368	73.24
12	21.998	1‐Hexacosanol, TMS derivative	C_29_H_62_OSi	454	3.38
13	23.231	*α*‐Tocopherol, TMS derivative	C_32_H_58_O_2_Si	502	0.54
14	23.927	Campesterol, TMS derivative	C_31_H_56_OSi	472	0.14
15	24.565	*β*‐Sitosterol, TMS derivative	C_32_H_58_OSi	486	22.39

**FIGURE 3:**
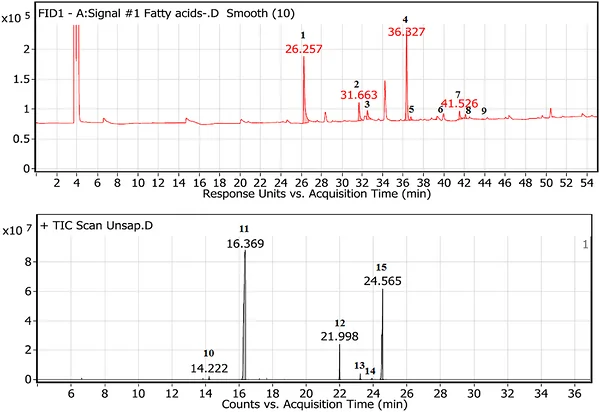
GC‐MS chromatogram of *n*‐hexane fraction from *C. althaeoides* aerial parts.

Analysis of the unsaponifiable fraction revealed six trimethylsilyl (TMS) derivatives, including two fatty alcohols (1‐hexadecanol and 1‐hexacosanol), one diterpene alcohol (phytol), two phytosterols (*β*‐sitosterol and campesterol), and *α*‐tocopherol (vitamin E). The identified constituents and their relative abundances are presented in Table 2 and Figure 3. Among these compounds, the TMS derivatives of phytol (73.24%) and *β*‐sitosterol (22.39%) were the most abundant components of the unsaponifiable fraction.

### 2.3 In Vitro Anticancer Activity

The cytotoxic potential of *C. althaeoides* fractions (EtOAc and *n*‐hexane) was assessed against three human cancer cell lines; HepG‐2 (liver carcinoma), MCF‐7 (breast adenocarcinoma), and HCT‐116 (colon carcinoma), using an LDH release assay to evaluate cell viability. Both fractions exhibited concentration‐dependent inhibitory effects on cancer cell proliferation (Figure S42). Among the tested samples, the EtOAc fraction demonstrated the most potent cytotoxicity with IC_50_ values of 5.8, 2.5, and 2.8 µg/mL against HepG‐2, MCF‐7, and HCT‐116, respectively (Table 3).

**TABLE 3:** In vitro anticancer activities (IC_50_) of the two plant fractions and Doxorubicin against three cancer and one normal human cell lines according to the LDH assay.

Sample test	IC_50_ (µg/mL) ± SD (*n* = 3)			
	HepG‐2	MCF‐7	HCT‐116	BJ‐1
EtOAc fraction	5.8 ± 0.4^*^	2.5 ± 0.1^*^	2.8 ± 0.1^*^	23.0 ± 2.4^*^
*n*‐hexane fraction	7.1 ± 0.5^*^	5.3 ± 0.3^*^	5.3 ± 0.3^*^	25.3 ± 2.2^*^
Doxorubicin (positive control)	4.8 ± 0.5^*^	5.6 ± 0.3^*^	6.5 ± 0.5^*^	32.1± 3.1^*^

* Statistical significance as compared to the control group at *p* < 0.05.

Notably, these values were generally lower than those of the reference chemotherapeutic doxorubicin (4.8, 5.6, and 6.5 µg/mL against HepG2, MCF‐7, and HCT‐116, respectively). These findings indicate the remarkable antiproliferative activity of the phenolic‐rich EtOAc fraction of *C. althaeoides* [12, 28, 33, 34, 36]. In contrast, *n*‐hexane fractions the *n*‐hexane fraction exhibited cytotoxic activity comparable to that of doxorubicin, with IC_50_ values of 7.1, 5.3, and 5.3 µg/mL against HepG2, MCF‐7, and HCT‐116, respectively (Table 3). The observed anticancer activity of the *n*‐hexane fraction may be attributed to the synergistic effects of its fatty acid constituents, together with *β*‐sitosterol and phytol, highlighting the potential contribution of these bioactive compounds to the anticancer properties of *C. althaeoides* [37, 38, 39, 40, 41].

Importantly, evaluation against the normal human fibroblast (BJ‐1) cell line revealed considerably higher IC_50_ values (23–32 µg/mL) for the tested samples, indicating selective cytotoxicity toward malignant cells. The EtOAc fraction exhibited selectivity index (SI) values of 3.9, 9.2, and 8.2 against HepG‐2, MCF‐7, and HCT‐116 cells, respectively, whereas the *n*‐hexane fraction showed SI values of 3.5, 4.7, and 4.7 against the corresponding cell lines. These findings suggest that both fractions possess selective anticancer activity while exhibiting relatively low cytotoxicity toward normal cells. The observed selectivity suggests that the bioactive constituents present in both the EtOAc and *n*‐hexane fractions preferentially interfere with molecular pathways essential for cancer cell survival while exerting minimal cytotoxic effects on non‐malignant cells, particularly in the case of the EtOAc fraction. This selective anticancer activity is consistent with previous reports demonstrating the tumor‐selective properties of plant‐derived phenolic compounds and other secondary metabolites [42, 43]. The superior cytotoxic performance of the EtOAc fraction can likely be attributed to its high content of phenolic constituents, including flavonoids and phenolic acids. These compounds have been extensively reported to induce apoptosis, promote cell cycle arrest, and suppress oncogenic signaling pathways through modulation of the PI3K/Akt and NF‐κB signaling cascades. Furthermore, their well‐established safety profile may account for the high selectivity indices observed for the EtOAc fraction, reinforcing its potential as a promising source of selective anticancer agents [44, 45].

### 2.4 In Vitro Antioxidant Activity

The antioxidant activities of the *n*‐hexane and EtOAc fractions of *C. althaeoides* were evaluated at concentrations of 0.0005, 0.001, and 0.005 mg/mL using the DPPH free radical scavenging assay. The results are presented in Figure 4. Both fractions demonstrated pronounced antioxidant activity in a concentration‐dependent manner, with the *n*‐hexane and EtOAc fractions achieving 90.12% and 97.22% DPPH radical scavenging, respectively, at the highest tested concentration (0.005 mg/mL). Notably, the antioxidant activity of the EtOAc fraction was comparable to that of the reference antioxidant, ascorbic acid. This remarkable free radical scavenging capacity is likely attributable to the high abundance of phenolic constituents, which are well recognized for their electron‐donating properties and their ability to modulate cellular redox homeostasis [28, 33, 36, 46].

**FIGURE 4:**
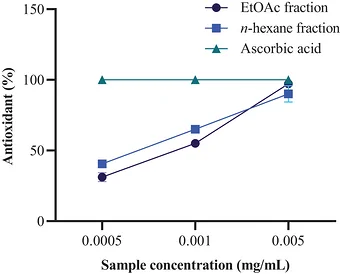
In vitro antioxidant activity (%) Mean ± SD (*n* = 3) of *C. althaeoides* aerial parts fractions and ascorbic acid using DPPH (Both the plant fractions and ascorbic acid showed statistical significance compared to the control group at *p* < 0.05).

Oxidative stress plays a central role in cancer development and progression by promoting the sustained generation of reactive oxygen species (ROS), which induce DNA damage and activate multiple pro‐survival signaling pathways, thereby contributing to tumor progression and resistance to chemotherapeutic agents [47]. The observed antioxidant activity exhibited by the EtOAc fraction of *C. althaeoides* suggests its potential role in cancer prevention and therapy through the attenuation of ROS‐induced genomic instability and the enhancement of cellular defense mechanisms against oxidative stress. This proposed mechanism is consistent with previous studies highlighting the protective effects of plant‐derived antioxidants against oxidative damage and their potential contribution to cancer chemoprevention and treatment [28, 33, 48].

### 2.5 In Vitro Anti‐Inflammatory Activity

The anti‐inflammatory activities of the EtOAc and *n*‐hexane fractions of *C. althaeoides* were evaluated using the human red blood cell (HRBC) membrane stabilization assay. As illustrated in Figure 5, both fractions exhibited moderate anti‐inflammatory activity, with the EtOAc and *n*‐hexane fractions producing 34.95% and 41.90% inhibition, respectively. Chronic inflammation is widely recognized as a key contributor to carcinogenesis, tumor progression, and metastasis through the sustained production of pro‐inflammatory mediators, including cyclooxygenase‐2 (COX‐2), tumor necrosis factor‐α (TNF‐α), and interleukin‐6 (IL‐6), as well as the persistent activation of the NF‐κB signaling pathway [49].

**FIGURE 5:**
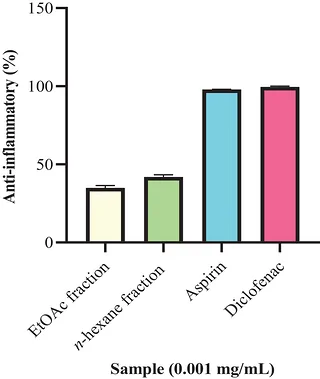
In vitro anti‐inflammatory activity (%) Mean ± SD (*n* = 3) of *C. althaeoides* aerial parts fractions, diclofenac sodium, and aspirin (Both the plant fractions and the standard drugs showed statistical significance compared to the control group at *p* < 0.05).

The observed anti‐inflammatory activity is consistent with the phytochemical composition of both fractions. In the EtOAc fraction, the effect may be attributed to its high content of phenolic compounds [12, 33, 36], whereas in the *n*‐hexane fraction it is likely associated with the presence of fatty acids, phytol, and phytosterols [50, 51, 52, 53]. These classes of bioactive compounds have been extensively reported to regulate immune responses and inhibit the production of major pro‐inflammatory cytokines. Although the anti‐inflammatory activity of the EtOAc and *n*‐hexane fractions was moderate, it may represent an important complementary mechanism underlying their overall anticancer potential. In addition to their cytotoxic and antioxidant properties, the ability of these fractions to modulate the tumor microenvironment may contribute to the suppression of chronic inflammation‐associated carcinogenesis, a process increasingly recognized as a critical factor in cancer initiation and progression [54].

Overall, the biological activities exhibited by the EtOAc and *n*‐hexane fractions of *C. althaeoides*, including antiproliferative, antioxidant, and anti‐inflammatory effects, suggest a multimodal mechanism of action that is consistent with current concepts in natural product‐based cancer therapy [55]. Among the two fractions, the EtOAc fraction, which is enriched in medium‐polarity phytochemicals, demonstrated the most pronounced cytotoxic activity, surpassing that of doxorubicin, while also exhibiting strong antioxidant activity and moderate anti‐inflammatory effects. These findings highlight the EtOAc fraction as a promising candidate for further investigation as a potential source of anticancer agents for both cancer prophylaxis and treatment.

Plant‐derived natural products remain an invaluable reservoir of bioactive compounds, many of which have successfully advanced into clinical application. The present findings further demonstrate that fractionation based on solvent polarity is an effective strategy for enriching chemically and biologically distinct constituents, thereby facilitating the identification of fractions with enhanced therapeutic potential [56].

### 2.6 In Silico Study

Anticancer therapy comprises a complex and growing group of drugs that belong to different classes and target different proteins, leading to inhibition of cell division. For example, microtubule stabilization such as taxol or microtubule antagonists (e.g., vinblastine) or a selective estrogen receptor modulator (SERM) such as tamoxifen. So, to understand and explore the mechanisms of the anticancer activity of our promising EtOAc fraction, the tentative identified compounds were tested against three pharmacophore hypothesis which built depending on the binding of tamoxifen analog (2EWP), taxol (5SYF), and vinblastine (8CLE) to their protein, respectively. In addition, the most fit compounds in each hypothesis (fit score above 1.5) were docked against the target protein with constraints that the tested compounds should bind at least one amino acid bound by the reference ligand. The selection of these pathways is based on two factors: First, the aim is to select enzymes that represent distinct mechanisms of action to test and demonstrate the multi‐target activity of the most potential extract, thereby making it difficult for drug resistance to develop. Second, the selection of enzymes is primarily based on the presence of some similarity in the pharmacophore between the selected compounds and the co‐crystalline ligands, or at least on previous reports indicating the ability of some compounds with a similar structure to act on these enzymes. Promising compounds from the pharmacophore and docking studies underwent an absorption, distribution, metabolism, excretion, and toxicity (ADME‐Tox) study to assess their drug likeness.

### 2.7 Pharmacophore, Docking, and ADME‐Tox Studies Results

Hypothesis 1 (hypothesis build depend on estrogen related receptor‐3 bind to tamoxifen analog): Six featured pharmacophore hypotheses were obtained (ADHRRR) (Figure 6a,b). Identified compounds were screened against this hypotheses, quercetin‐3‐*O*‐pentoside, and kaempferol‐3‐*O*‐pentoside showed the highest fitting among our compounds (fit score 2.160 and 1.914, respectively), both compounds showed four matched ligand sites (one acceptor, one donor and two rings AD‐R‐R) (Figure 6c,d).

**FIGURE 6:**
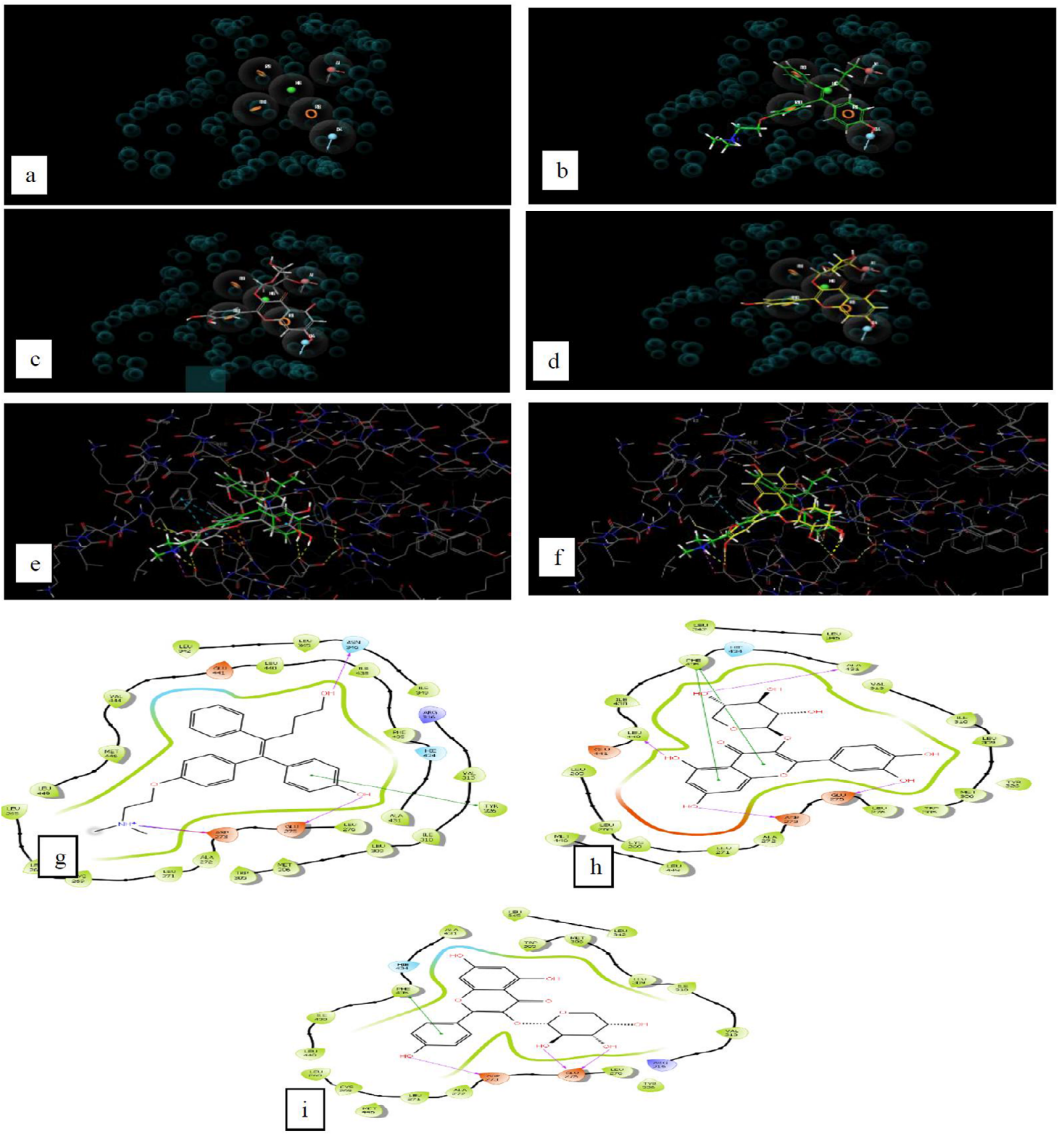
(a) Six featured pharmacophore hypotheses build depend on Estrogen Related Receptor‐3 (ERR‐3) bind to tamoxifen analog (ADHRRR), (b) Tamoxifen analogue (green) fit on hypothesis 1 (ADHRRR), (c) Quercetin‐3‐*O*‐pentoside (gray) fit in hypothesis 1 (AD‐R‐R), (d) Kaempferol‐3‐*O*‐pentoside (yellow) fit in hypothesis 1 (AD‐R‐R), (e) 3D for the orientation of tamoxifen analog (green) and quercetin‐3‐*O*‐pentoside (gray) in ERR‐3, (f) 3D for the orientation of tamoxifen analog (green) and kaempferol‐3‐*O*‐pentoside (yellow) in ERR‐3, (g) 2D image for tamoxifen analog in ERR‐3, (h) 2D image for quercetin‐3‐*O*‐pentoside in ERR‐3, (i) 2D image for kaempferol‐3‐*O*‐pentoside in ERR‐3.

Docking 1 (docking on estrogen related receptor‐3): Quercetin‐3‐*O*‐pentoside and kaempferol‐3‐*O*‐pentoside were docked on estrogen related receptor. Both compounds showed good docking score (−11.497 and −10.851, respectively). Both compounds showed orientation in the active site similar to tamoxfen analogue with the ability to bind by similar way to some extent, as the two compounds can form hydrogen bonds with GLU 275 and ASP 273 through OH groups, while tamoxifen binds to GLU 275 and ASP 273 by OH and NH groups, respectively (Figure 6e–i).

Hypothesis 2 (built depend on taxol‐stabilized microtubule): Six featured hypotheses were obtained (AADRRR) (Figure 7a,b). Identified compounds were screened against this hypothesis, rutin showed the highest number of features (five features from six, AAD‐RR) with fit score 1.813 (Figure 7c).

**FIGURE 7:**
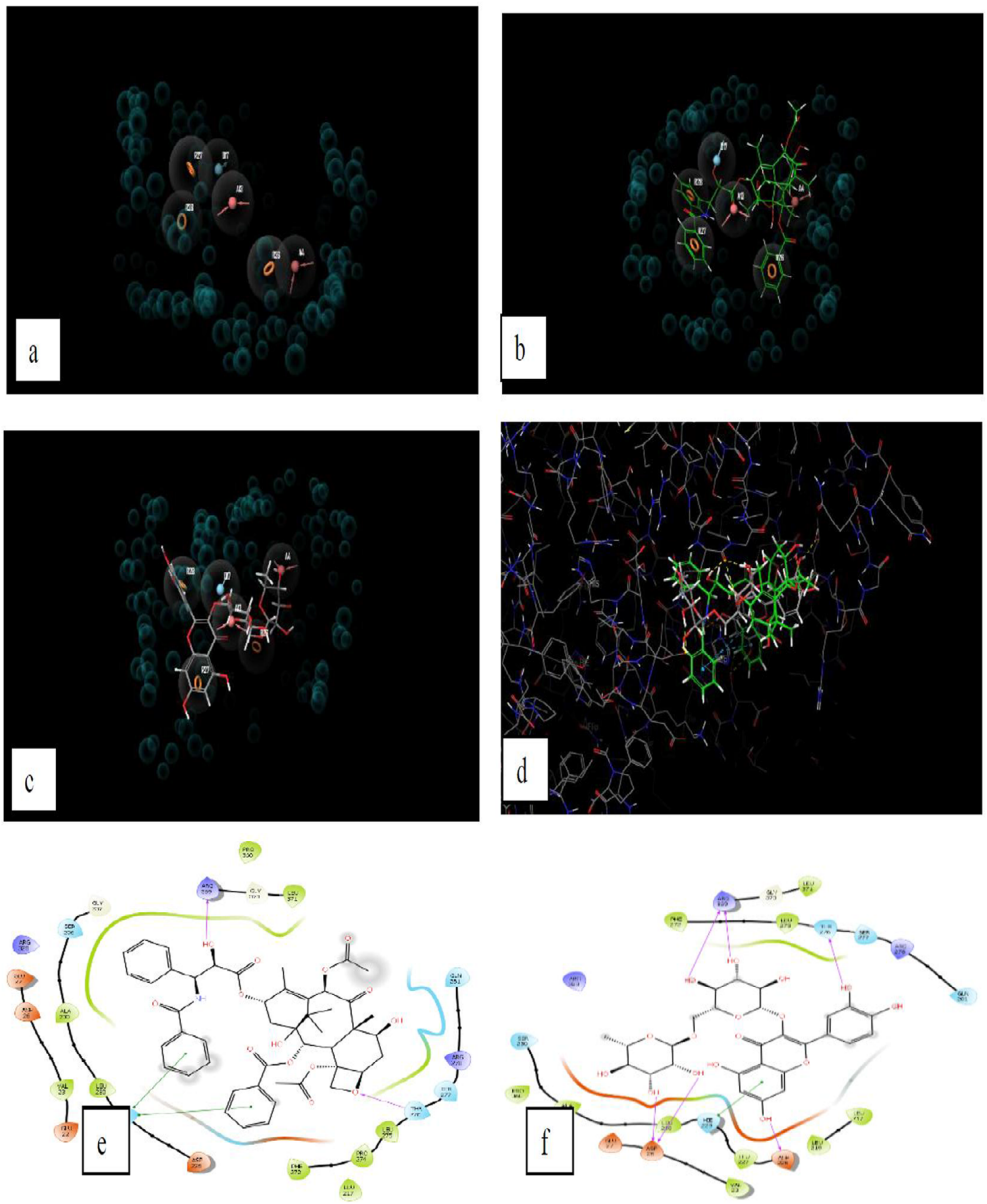
(a) Six featured pharmacophore hypotheses build depend on Taxol‐stabilized microtubule (AADRRR), (b) Taxol (green) fit on hypothesis 2 (AADRRR), (c) Rutin (gray) fit on hypothesis 2 (AAD‐RR), (d) 3D for the orientation of taxol (green) and rutin (gray) in microtubule, (e) 2D image for taxol in microtubule, (f) 2D image for rutin in microtubule.

Docking 2 (docking on taxol‐stabilized microtubule): Rutin was docked on microtubule. Rutin showed good docking score ‐10.801 and also showed orientation in the active site like taxol with the ability to bind by similar way to some extent. Rutin forms a hydrogen bond with THR 276 through the OH group (hydrogen bond donor) and one π‐π stacking bond with HIE 229 through the aromatic ring, while Taxol forms a hydrogen bond with THR 276 through the OH group (hydrogen bond acceptor) and two π‐π stacking bonds with HIE 229 through two aromatic rings (Figure 7d–f).

Hypothesis 3 (build depend on vinblastine bound to tubulin): Four featured hypotheses were obtained (DDRR) (Figure 8a,b). Identified compounds were screened against this hypotheses, quercetin‐3‐*O*‐glucoside showed the highest number of features (four features from four, DDRR) with fit score 1.755 (Figure 8c).

**FIGURE 8:**
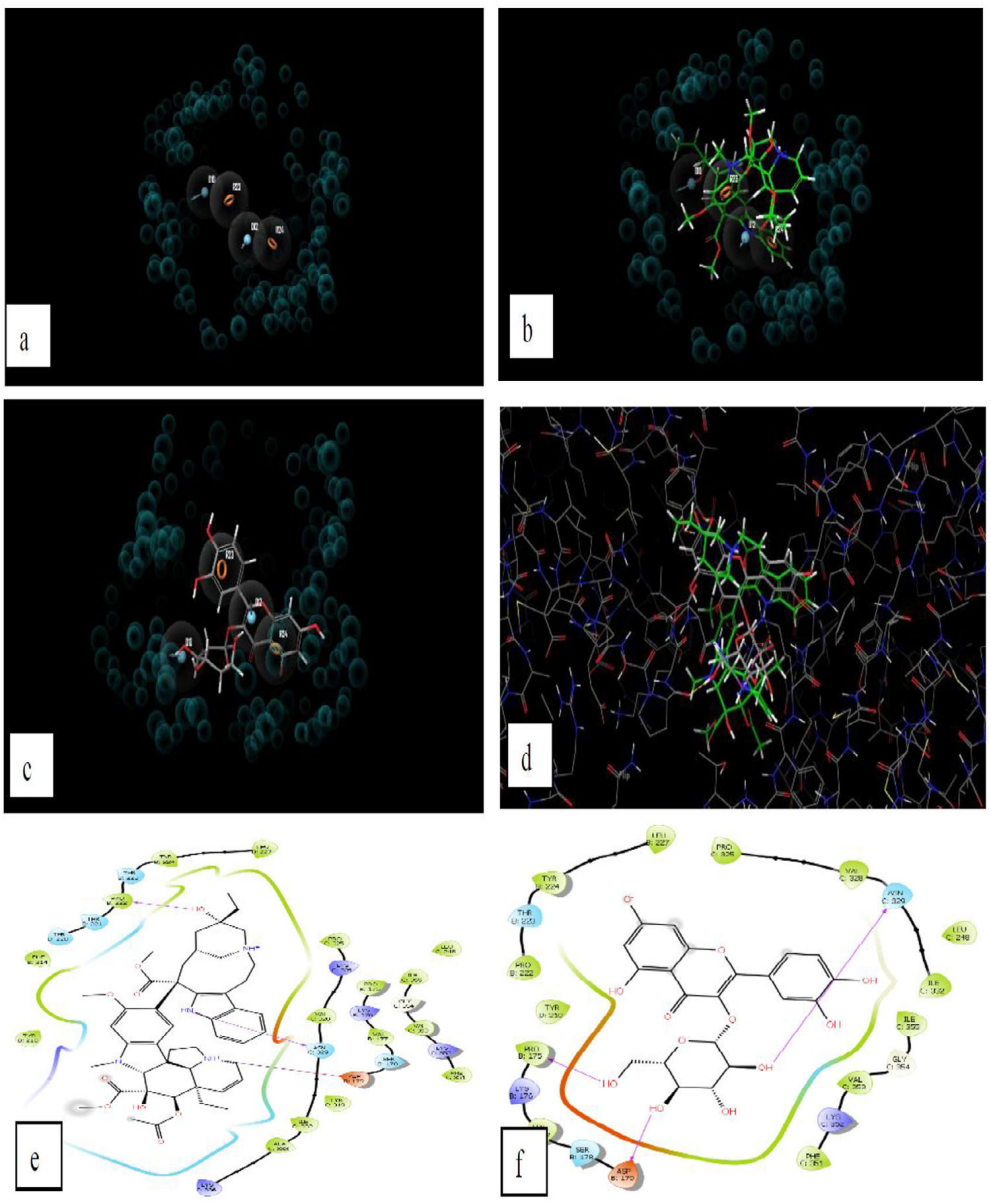
(a) Four featured pharmacophore hypotheses build depend on Vinblastine bound to tubulin (DDRR), (b) vinblastine (green) fit on hypothesis 3 (DDRR), (c) Quercetin‐3‐*O*‐glucoside (gray) fit on hypothesis 3 (DDRR), (d) 3D for the orientation of vinblastine (green) and quercetin‐3‐*O*‐glucoside (gray) in tubulin, (e) 2D image for vinblastine in tubulin, (f) 2D image for quercetin‐3‐*O*‐glucoside in tubulin.

Docking 3 (docking on vinblastine bound to tubulin): Quercetin‐3‐*O*‐glucoside was docked on tubulin. Quercetin‐3‐*O*‐glucoside showed good docking score −7.113. Quercetin‐3‐*O*‐glucoside also showed orientation in the active site like vinblastine with the ability to bind by similar way to some extent. Quercetin‐3‐*O*‐glucoside forms a hydrogen bond with ASP 170 through the OH group, while vinblastine forms an ionic bond with ASP 170 through the NH group (Figure 8d–f).

ADME‐Tox study: Quercetin‐3‐*O*‐pentoside, kaempferol‐3‐*O*‐pentoside, rutin, and quercetin‐3‐*O*‐glucoside were subjected to an ADME/Tox study. The most important properties, including metabolism, percent human oral absorption, rule of five, rule of three, and QPlogS, are summarized in Table 4. The results showed that although the four compounds had poor oral absorption, they still show potential for drug likeness, as all the compounds showed good water solubility (QPlogS above ‐6) and all (except rutin) showed two or fewer violations to the rule of three and the rule of five.

**TABLE 4:** The important ADME/Tox properties of quercetin‐3‐*O*‐pentoside, kaempferol‐3‐*O*‐pentoside, rutin and quercetin‐3‐*O*‐glucoside.

ADME/Tox properties					
Compounds	metab^*^	Percent human oral absorption	Rule of five	Rule of three	QPlogS
Quercetin‐3‐*O*‐pentoside	7	10.355	2	2	−2.815
kaempferol ‐3‐*O*‐pentoside	6	33.569	1	1	−2.795
Rutin	10	0	3	2	−2.138
Quercetin‐3‐*O*‐glucoside	8	0	2	2	−2.343

* Number of possible metabolic liabilities.

## 3 Conclusions

This study highlights the potential of *C. althaeoides* growing in Egypt to be a source of plant‐based natural products for prophylaxis against and treating different types of cancers (liver, breast, and colon), while the phytochemical and in silico studies showed the possible chemical profile and mechanisms responsible for these activities. The current findings showed for the first time that both *n*‐hexane and EtOAc fractions (successively fractionated from 70% ethanolic extract of *C. althaeoides* aerial parts) had potent cytotoxic activity against the tested cancer cell lines (with EtOAc fraction showed in general lower IC_50_ than the standard doxorubicin) with good selectivity index (higher IC_50_ against normal human cell line), significant antioxidant and moderate anti‐inflammatory effects. These activities together emphasize the possible role for these two fractions to prevent and control the trigger factors for cancers (oxidative stress and inflammation) and, at the same time, kill, or induce apoptosis for any mutated cancer cell. The phytochemical investigation for EtOAc fraction (LC‐ESI‐MS/MS) and *n*‐hexane fraction (GC‐MS) showed the chemical profile advantages for these fractions responsible for these activities. While the in silico studies showed that EtOAc fraction contains phyto‐compounds that can act as anticancer through different mechanisms, for example, they can act on Estrogen Related Receptor through quercetin‐3‐*O*‐pentoside and kaempferol‐3‐*O*‐pentoside, or they can act as microtubule stabilization through rutin or on tubulin through quercetin‐3‐*O*‐glucoside. Future work integrating phytochemical isolation and purification with mechanistic studies (e.g., apoptosis markers, cell cycle analysis, gene expression, and animal or cellular models) will be critical to elucidate the molecular underpinnings of the observed activities fully.

## 4 Experimental Section

### 4.1 Plant Material

The aerial parts of *Convolvulus althaeoides* L. were gathered in April 2019 from the Northwestern coastal of Egypt (Wadi Habis, Mersa matrouh governorate) (GPS coordinates: 31°22′81″ N; 27°3′54″ E). The plant sample was kindly authenticated by Dr. Omran Ghaly, Doctor Researcher of plant ecology at Desert Research Center, Egypt. A voucher specimen (CAIH‐29‐12‐2019‐R) has been deposited at the herbarium of Desert Research Center, Egypt.

### 4.2 Extraction and Fractionation

The plant material was air‐dried in shade and processed into a finely ground powder. 300 g of *C. althaeoides* aerial parts were subsequently macerated in 70% ethanol (70% EtOH, 5 times × each 800 mL, 24 h). The aqueous ethanolic extract was filtered, combined, and then concentrated using rotavapor at 50°C, yielding 26 g of the total dried extract. 24 g of the 70% EtOH extract was next sequentially fractionated by liquid‐liquid partition extraction with *n*‐hexane and ethyl acetate (EtOAc). These two separated fractions were then concentrated under reduced pressure until dryness, resulting in 4.9 g *n*‐hexane fraction, 12.5 g EtOAc fraction, and a residue (remaining aqueous fraction) weighing 6.6 g.

### 4.3 LC‐ESI‐MS/MS Analysis of *C. althaeoides* EtOAc Fraction

The metabolic profile the active EtOAc fraction was performed using liquid chromatography–electrospray ionization–tandem mass spectrometry (LC‐ESI‐MS/MS) [57] with an ExionLC AC system for separation and SCIEX Triple Quad 5500+ MS/MS system equipped with an electrospray ionization (ESI) for detection of the separated peaks. An Ascentis Express 90 Å C18 Column (2.1 × 150 mm, 2.7 µm) was used. The gradient elution was carried using a mobile phase consisting of two eluents A: 5 mM ammonium formate pH 8; B: acetonitrile (LC grade). The gradient method was performed as follows: 5% B at 0‐1 min, 5–100% B from 1‐20 min, 100% B from 20‐25 min, 5% at 25.01, and 5% from 25.01–30 min. The flow rate was 0.3 mL/min and the injection volume was 5 µl. For MS/MS analysis, negative ionization mode was applied with a scan (EMS‐IDA‐EPI) from 100 to 1000 Da for MS1 with the following parameters: curtain gas 25 psi, ion spray voltage −4500, source temperature, 500°C, ion source gas 1 & 2 were 45 psi ranged from 50 to 1000 Da for MS2 with a declustering potential −80, and collision energy −35. The MS spectra of our sample were processed using SCIEX PeakView software and the resulted peaks were tentatively speculated by comparing their MS/MS fragmentation pattern with the reported data as well as mass spectra databases.

### 4.4 GC‐MS Analysis of *C. althaeoides n*‐Hexane Fraction

The plant *n*‐hexane fraction was initially divided into two samples. The first one was saponified with ethanolic KOH, and then acidified with HCl, and the free fatty acids were extracted by *n*‐hexane, then evaporated under nitrogen. After that, fatty acid methyl esters (FAME) are produced by an acidic catalyst between fats and methanol in the presence of boron trifluoride‐methanol solution 7% in methanol at 100°C for 45 min and injected in dichloromethane. On the other hand, the second sample was saponified with ethanolic potassium hydroxide; the unsaponifiable matter was extracted with *n*‐hexane and evaporated at 40°C. Then, the unsaponifiable fraction was subjected to silylation by extraction with ether and petroleum ether (1:1). Then mixed with 50 µL of the silylation reagent, which consists of bis(trimethylsilyl)trifluoroacetamide (BSTFA) and trimethylchlorosilane (TMCS) 99:1, and then 50 µL pyridine were added for derivatization of sample functional groups to trimethylsilyl groups (abbreviated TMS) before GC analysis. The GC model 7890B from Agilent Technologies was equipped with a flame ionization spectrometer detector (5977A). Separation of the saponifiable matter (FAME) was achieved using a Zebron ZB‐FAME column (60 m × 0.25 mm internal diameter × 0.25 µm film thickness). Analyses were carried out using hydrogen as the carrier gas at a flow rate of 1.8 mL/min in split mode (1:50), with an injection volume of 1 µL, and the following temperature program: 100°C for 3 min, then rising at 2.5°C/min to 240°C and held for 10 min. The injector and detector (FID) were held at 250°C and 285°C, respectively. However, unsaponifiable matter (silylated derivatives) was analyzed using an HP‐5MS column (30 m × 0.25 mm internal diameter and 0.25 µm film thickness). Analyses were carried out using Hydrogen as the carrier gas at a flow rate of 1.0 mL/min at a splitless, injection volume of 1 µl and the following temperature program: 60°C for 1 min; rising at 10°C /min to 320°C and held for 10 min. The injector and detector were held at 300°C, 320°C. Mass spectra were obtained by electron ionization (EI) at 70 eV, using a spectral range of *m/z* 50‐800 and a solvent delay of 3.9 min. The mass temperature was 230°C, and the Quad was 150°C. Identification of different constituents was determined by comparing the spectrum fragmentation pattern with those stored in the Wiley and NIST Mass Spectral Library data [17].

### 4.5 In Vitro Anticancer Activity (Lactate Dehydrogenase LDH Assay)

Roswell Park Memorial Institute (RPMI) 1640 medium was purchased from Sigma Chem. Co. (St. Louis, MO, USA). Fetal bovine serum (FBS) and fetal calf serum (FCS) were purchased from Gibco, UK. Dimethyl sulfoxide (DMSO) and methanol were of HPLC grade, and all other reagents and chemicals were of analytical reagent grade. Human liver carcinoma (HepG‐2, RRID:CVCL_0027), human colorectal carcinoma (HCT116, RRID:CVCL_0291), human breast adenocarcinoma (MCF‐7, RRID:CVCL_0031), and the normal human skin fibroblast (BJ‐1, RRID:CVCL_3653) cell lines were purchased from the American Type Culture Collection (Rockville, MD, USA) and obtained from the National Cancer Institute (NCI), Cairo University, Egypt. These cancer cell lines were maintained in RPMI‐1640 medium which was supplemented with 10% heat‐inactivated FBS, 100 U/mL penicillin and 100 U/mL streptomycin. The cells were grown at 37°C in a humidified atmosphere of 5% CO_2_. All experiments were conducted thrice in triplicate (*n* = 3). All the values were represented as means ± SD. To determine the effect of each plant fraction (*n*‐hexane and EtOAc) on membrane permeability in HepG2, MCF‐7, and HCT‐116 cancer cell lines as well as BJ‐1 normal cell line, a lactate dehydrogenase (LDH) release assay was used [58]. The cells were seeded in 24‐well culture plates at a density of 1 × 10^4^ cells/well in 500 µL volume and allowed to grow for 18 h before treatment. After treatment with a series of different concentrations of each compound or doxorubicin (positive control), the plates were incubated for 48 h. Then, the supernatant (40 µL) was transferred to a new 96 well to determine LDH release and 6% triton X‐100 (40 µL) was added to the original plate for determination of total LDH. An aliquot of 0.1 M potassium phosphate buffer (100 µL, pH 7.5) containing 4.6 mM pyruvic acid was mixed to the supernatant using repeated pipetting. Then, 0.1 M potassium phosphate buffer (100 µL, pH 7.5) containing 0.4 mg/mL reduced β‐NADH was added to the wells. The kinetic changes were read for 1 min using ELISA microplate reader in absorbance at wavelength 340 nm. This procedure was repeated with 40 µL of the total cell lysate to determine total LDH. The percentage of LDH release was determined by dividing the LDH released into the media by the total LDH following cell lysis in the same well.

### 4.6 Antioxidant Activity Using DPPH Radical Scavenging Assay

The DPPH (1–diphenyl–2–picrylhydrazyl) scavenging activity of the samples was determined quantitatively by the method [59]. A 500 µl of ethanolic DPPH solution (0.4 mmol) was mixed vigorously with 500 µL of each sample (with different concentrations, initially 0.01 g/mL), or water (as a control) and incubated at 37°C in the dark for 1 h. The absorbance of the mixture was measured spectrophotometrically at 517 nm. The scavenging activity was calculated according to the following equation:

$$\%\;\text{Scavenging activity}=1-\left(\frac{As-Ab}{Ac}\right)\times 100$$

Whereas *Ab*, *Ac*, and *As* are the absorbance of the blank (ethanol and sample), the control (DPPH and deionized water) and the sample (DPPH and sample), respectively. This experiment was conducted duplicate; all values are expressed as means ± standard deviation (SD) and compared to the control (ascorbic acid). Statistical analysis is done as previously mentioned.

### 4.7 Anti‐Inflammatory Activity Using Human Red Blood Cell Stabilization Method

The human red blood cell (HRBC) membrane stabilization method was used to study the in vitro anti‐inflammatory activity of the samples [60]. Blood was collected from one healthy volunteer. The collected blood was mixed with equal volume of sterilized Alsever solution (2% dextrose, 0.8% sodium citrate, 0.5% citric acid and 0.42% sodium chloride in water). The blood was then centrifuged at 3000 rpm for 20 min and packed cells were separated. The packed cells were washed with isosaline (0.85%, pH 7.2) and a 10% v/v suspension was made with isosaline. This HRBC suspension was used for the estimation of anti‐inflammatory property. One milliliter of plant fractions, diclofenac sodium and aspirin were separately mixed with 1 mL of phosphate buffer (0.15 M, pH 7.4), 2 mL of hypo saline (0.36%) and 0.5 mL of HRBC suspension. Instead of sample, 2 mL distilled water was used as the negative control. All the assay mixture was incubated at 37°C for 30 min and centrifuged at 3000 rpm for 20 min. The supernatant liquid was decanted and the hemoglobin content in the supernatant solution was estimated using spectrophotometer at 560 nm. Percentage hemolysis was estimated by assuming the hemolysis produced in the negative control as 100%.

The percentage of HRBC membrane stabilization or protection was calculated by using the following formula:

$$\%\;\text{Protection}=100-\left(\frac{\mathrm{OD}\;\text{sample}}{\mathrm{OD}\;\text{control}}\right)\times 100$$

### 4.8 Statistical Analysis

All experiments were conducted in triplicate (*n* = 3). All the values were represented as mean ± SD. Significant differences between the means of parameters as well as IC_50_ values were determined by one‐way ANOVA and probit analysis using Graphpad prism 8 and SPSS software programs (SPSS Inc., Chicago, IL).

### 4.9 In Silico Study

All in silico work was done by using Maestro 11.4 (Schrödinger Release 2017‐4: Maestro; Schrödinger, LLC: New York, NY, USA, 2017). Crystal structures 2EWP [61], 5SYF [62], and 8CLE [63] were prepared using Maestro tool protein preparation wizard. A grid box centered on the native ligand was used to define the binding pocket of the protein. Extra precision (XP) setting had been used during docking. The docking protocol was validated by re‐docking the co‐crystallized ligands to the enzymes during docking of the identified compounds, and the RMSD was calculated, and the overlay of co‐crystallized versus re‐docked was evaluated. The RMSD was calculated during Glide docking by choosing core pattern comparison. For the ADME‐Tox study, QikProp was used.

## Author Contributions

**Ahmed R. Hassan**: conceptualization, methodology, investigation, data curation, writing – original draft, writing – review & editing. **Shaimaa Negm El‐Dein**: methodology, investigation, and data curation, writing – review & editing. **Amany M. Korkor**: methodology, investigation, and data curation, writing – review & editing. **Nourelhoda F. Hassan**: methodology, investigation, data curation, writing – review & editing. **Ahmed A.M.A. Selim**: conceptualization, methodology, investigation, data curation, writing – original draft, writing – review & editing.

## Ethical Approval and Consent to Participate Declaration

Peripheral blood was collected from a healthy volunteer that consented to the use of the blood sample for the conducted anti‐inflammatory assays and the publication of the resulting data. Informed written consent of all participants was obtained. Concerning tumor specimens were obtained from the National Cancer Institute to be used exclusively for an *ex vivo* antitumor assay. No human participants were recruited, and no additional intervention or sample collection was performed for research purposes. According to the regulations and policies of the National Research Centre Ethics Committee, ethical approval was not required for this type of *ex vivo* study using previously obtained tumor specimens.

## Conflicts of Interest

The authors declare no conflicts of interest.

## Supporting information




**Supporting File 1**: cbdv71792‐sup‐0001‐SuppMat.docx
Supporting Information for this article is available on the under https://doi.org/10.1002/cbdv.71792.

## Data Availability

The data that support the findings of this study are available in the supplementary material of this article.
