# Supplementary material for: Convolvulus althaeoides as a Potential Natural Source in Cancer Prophylaxis and Treatment: LC‐ESI‐MS/MS, GC‐MS, In Vitro, and In Silico Investigations
Source: Chem Biodivers. 2026 Sep 30;23(10):e71792. doi: 10.1002/cbdv.71792 (PMC13626262; doi:10.1002/cbdv.71792)
Supplement: Supplementary file 1 [file CBDV-23-e71792-s001.docx]

**Supplementary Material**

***Convolvulus althaeoides* as a potential natural source in cancer prophylaxis and treatment: LC-ESI-MS/MS, GC-MS, *in vitro*, and *in silico* investigations**

Ahmed R. Hassan^a^*, Shaimaa Negm El-Dein^b^, Amany M. Korkor^b^, Nourelhoda F. Hassan^b^, Ahmed A.M.A. Selim^a^

^a^ Medicinal and Aromatic Plants Department, Desert Research Center, Cairo 11753, Egypt

^b^ Department of Pharmacognosy, Faculty of Pharmacy (for Girls), Al-Azhar University, Nasr City 11651, Cairo, Egypt

^*^ Corresponding author: [drarahdrc@gmail.com](mailto:drarahdrc@gmail.com)**,** [ahmedhassan@drc.gov.eg](mailto:ahmedhassan@drc.gov.eg)

**Abstract**

The current study aims to assess the potential relevance of the successive fractions, *n*-hexane and ethyl acetate (EtOAc) of *Convolvulus althaeoides* in cancer management. Our study demonstrated significant *in vitro* cytotoxic activity with IC_50_ values of 5.8, 2.5, and 2.8 for EtOAc and 7.1, 5.3, and 5.3 µg/mL for *n*-hexane against HepG-2, MCF-7, and HCT-116, respectively, comparable to doxorubicin (4.8, 5.6, and 6.5 µg/mL), with a higher IC_50_ against normal human cell line. EtOAc and *n*-hexane fractions exhibited substantial antioxidant using DPPH (97.22% and 90.12%, respectively) and moderate anti-inflammatory properties by HRBC method (34.95% and 41.9%, respectively). LC-ESI-MS/MS analysis of EtOAc fraction revealed 41 peaks with varied chemical structures, indicating its richness of phenolic compounds. GC-MS of *n*-hexane fraction indicated an abundance of noteworthy fatty acids, phytol, and phytosterols. The *in silico* results showed good pharmacophore features and docking scores for some of the identified compounds on ERR-3 (quercetin-3-*O*-pentoside -11.497), microtubule (rutin -10.801), and tubulin (quercetin-3-*O*-glucoside -7.113).

**Keywords:** *Convolvulus althaeoides*, anticancer activity, LC-ESI-MS/MS, GC-MS, *in silico* study

**Fig. S1:** ESI**^−^–**MS of peak 1 detected in LC-ESI-MS/MS chromatogram of *C. althaeoides* EtOAc fraction

**Fig. S2:** ESI**^−^–**MS of peak 2 detected in LC-ESI-MS/MS chromatogram of *C. althaeoides* EtOAc fraction

**Fig. S3:** ESI**^−^–**MS of peak 3 detected in LC-ESI-MS/MS chromatogram of *C. althaeoides* EtOAc fraction

**Fig. S4:** ESI**^−^–**MS of peak 4 detected in LC-ESI-MS/MS chromatogram of *C. althaeoides* EtOAc fraction

**Fig. S5:** ESI**^−^–**MS of peak 5 detected in LC-ESI-MS/MS chromatogram of *C. althaeoides* EtOAc fraction

**Fig. S6:** ESI**^−^–**MS of peak 6 detected in LC-ESI-MS/MS chromatogram of *C. althaeoides* EtOAc fraction

**Fig. S7:** ESI**^−^–**MS of peak 7 detected in LC-ESI-MS/MS chromatogram of *C. althaeoides* EtOAc fraction

**Fig. S8:** ESI**^−^–**MS of peak 8 detected in LC-ESI-MS/MS chromatogram of *C. althaeoides* EtOAc fraction

**Fig. S9:** ESI**^−^–**MS of peak 9 detected in LC-ESI-MS/MS chromatogram of *C. althaeoides* EtOAc fraction

**Fig. S10:** ESI**^−^–**MS of peak 10 detected in LC-ESI-MS/MS chromatogram of *C. althaeoides* EtOAc fraction

**Fig. S11:** ESI**^−^–**MS of peak 11 detected in LC-ESI-MS/MS chromatogram of *C. althaeoides* EtOAc fraction

**Fig. S12:** ESI**^−^–**MS of peak 12 detected in LC-ESI-MS/MS chromatogram of *C. althaeoides* EtOAc fraction

**Fig. S13:** ESI**^−^–**MS of peak 13 detected in LC-ESI-MS/MS chromatogram of *C. althaeoides* EtOAc fraction

**Fig. S14:** ESI**^−^–**MS of peak 14 detected in LC-ESI-MS/MS chromatogram of *C. althaeoides* EtOAc fraction

**Fig. S15:** ESI**^−^–**MS of peak 15 detected in LC-ESI-MS/MS chromatogram of *C. althaeoides* EtOAc fraction

**Fig. S16:** ESI**^−^–**MS of peak 16 detected in LC-ESI-MS/MS chromatogram of *C. althaeoides* EtOAc fraction

**Fig. S17:** ESI**^−^–**MS of peak 17 detected in LC-ESI-MS/MS chromatogram of *C. althaeoides* EtOAc fraction

**Fig. S18:** ESI**^−^–**MS of peak 18 detected in LC-ESI-MS/MS chromatogram of *C. althaeoides* EtOAc fraction

**Fig. S19:** ESI**^−^–**MS of peak 19 detected in LC-ESI-MS/MS chromatogram of *C. althaeoides* EtOAc fraction

**Fig. S20:** ESI**^−^–**MS of peak 20 detected in LC-ESI-MS/MS chromatogram of *C. althaeoides* EtOAc fraction

**Fig. S21:** ESI**^−^–**MS of peak 21 detected in LC-ESI-MS/MS chromatogram of *C. althaeoides* EtOAc fraction

**Fig. S22:** ESI**^−^–**MS of peak 22 detected in LC-ESI-MS/MS chromatogram of *C. althaeoides* EtOAc fraction

**Fig. S23:** ESI**^−^–**MS of peak 23 detected in LC-ESI-MS/MS chromatogram of *C. althaeoides* EtOAc fraction

**Fig. S24:** ESI**^−^–**MS of peak 24 detected in LC-ESI-MS/MS chromatogram of *C. althaeoides* EtOAc fraction

**Fig. S25:** ESI**^−^–**MS of peak 25 detected in LC-ESI-MS/MS chromatogram of *C. althaeoides* EtOAc fraction

**Fig. S26:** ESI**^−^–**MS of peak 26 detected in LC-ESI-MS/MS chromatogram of *C. althaeoides* EtOAc fraction

**Fig. S27:** ESI**^−^–**MS of peak 27 detected in LC-ESI-MS/MS chromatogram of *C. althaeoides* EtOAc fraction

**Fig. S28:** ESI**^−^–**MS of peak 28 detected in LC-ESI-MS/MS chromatogram of *C. althaeoides* EtOAc fraction

**Fig. S29:** ESI**^−^–**MS of peak 29 detected in LC-ESI-MS/MS chromatogram of *C. althaeoides* EtOAc fraction

**Fig. S30:** ESI**^−^–**MS of peak 30 detected in LC-ESI-MS/MS chromatogram of *C. althaeoides* EtOAc fraction

**Fig. S31:** ESI**^−^–**MS of peak 31 detected in LC-ESI-MS/MS chromatogram of *C. althaeoides* EtOAc fraction

**Fig. S32:** ESI**^−^–**MS of peak 32 detected in LC-ESI-MS/MS chromatogram of *C. althaeoides* EtOAc fraction

**Fig. S33:** ESI**^−^–**MS of peak 33 detected in LC-ESI-MS/MS chromatogram of *C. althaeoides* EtOAc fraction

**Fig. S34:** ESI**^−^–**MS of peak 34 detected in LC-ESI-MS/MS chromatogram of *C. althaeoides* EtOAc fraction

**Fig. S35:** ESI**^−^–**MS of peak 35 detected in LC-ESI-MS/MS chromatogram of *C. althaeoides* EtOAc fraction

**Fig. S36:** ESI**^−^–**MS of peak 36 detected in LC-ESI-MS/MS chromatogram of *C. althaeoides* EtOAc fraction

**Fig. S37:** ESI**^−^–**MS of peak 37 detected in LC-ESI-MS/MS chromatogram of *C. althaeoides* EtOAc fraction

**Fig. S38:** ESI**^−^–**MS of peak 38 detected in LC-ESI-MS/MS chromatogram of *C. althaeoides* EtOAc fraction

**Fig. S39:** ESI**^−^–**MS of peak 39 detected in LC-ESI-MS/MS chromatogram of *C. althaeoides* EtOAc fraction

**Fig. S40:** ESI**^−^–**MS of peak 40 detected in LC-ESI-MS/MS chromatogram of *C. althaeoides* EtOAc fraction

**Fig. S41:** ESI**^−^–**MS of peak 41 detected in LC-ESI-MS/MS chromatogram of *C. althaeoides* EtOAc fraction

**Fig. S42:** Dose dependent *in vitro* antiproliferative data of the two plant fractions and Doxorubicin against three cancer and one normal human cell lines according to the LDH assay after 48 h of exposure
